# Nanoscale Electrical Potential and Roughness of a Calcium Phosphate Surface Promotes the Osteogenic Phenotype of Stromal Cells

**DOI:** 10.3390/ma11060978

**Published:** 2018-06-09

**Authors:** Igor A. Khlusov, Yuri Dekhtyar, Yurii P. Sharkeev, Vladimir F. Pichugin, Marina Y. Khlusova, Nataliya Polyaka, Fjodors Tjulkins, Viktorija Vendinya, Elena V. Legostaeva, Larisa S. Litvinova, Valeria V. Shupletsova, Olga G. Khaziakhmatova, Kristina A. Yurova, Konstantin A. Prosolov

**Affiliations:** 1 Research School of Chemistry & Applied Biomedical Sciences, National Research Tomsk Polytechnic University, Tomsk 634050, Russia; 2 Basic Laboratory of Immunology and Cell Biotechnology, Immanuel Kant Baltic Federal University, Kaliningrad 236041, Russia; larisalitvinova@yandex.ru (L.S.L.); vshupletsova@mail.ru (V.V.S.); hazik36@mail.ru (O.G.K.); kristina_kofanova@mail.ru (K.A.Y.); 3 Institute of Biomedical Engineering and Nanotechnologies, Riga Technical University, Riga LV-1658, Latvia; dekhtyar@latnet.lv (Y.D.); natalija.polaka@latnet.lv (N.P.); Kgam@inbox.lv (F.T.); vicken@inbox.lv (V.V.); 4 Research School of High-Energy Physics, National Research Tomsk Polytechnic University, Tomsk 634050, Russia; sharkeev@ispms.tsc.ru (Y.P.S.); pichugin@tpu.ru (V.F.P.); konstprosolov@gmail.com (K.A.P.); 5 Institute of Strength Physics and Materials Science of SB RAS, Tomsk 634055, Russia; lego@ispms.tsc.ru; 6 Department of Pathophysiology, Siberian State Medical University, Tomsk 634050, Russia; uchsovet@ssmu.ru

**Keywords:** surface roughness, surface electrical potential, micro- and nanoscale, human mesenchymal cells, osteocalcin, alkaline phosphatase, in vitro

## Abstract

Mesenchymal stem cells (MSCs) and osteoblasts respond to the surface electrical charge and topography of biomaterials. This work focuses on the connection between the roughness of calcium phosphate (CP) surfaces and their electrical potential (EP) at the micro- and nanoscales and the possible role of these parameters in jointly affecting human MSC osteogenic differentiation and maturation in vitro. A microarc CP coating was deposited on titanium substrates and characterized at the micro- and nanoscale. Human adult adipose-derived MSCs (hAMSCs) or prenatal stromal cells from the human lung (HLPSCs) were cultured on the CP surface to estimate MSC behavior. The roughness, nonuniform charge polarity, and EP of CP microarc coatings on a titanium substrate were shown to affect the osteogenic differentiation and maturation of hAMSCs and HLPSCs in vitro. The surface EP induced by the negative charge increased with increasing surface roughness at the microscale. The surface relief at the nanoscale had an impact on the sign of the EP. Negative electrical charges were mainly located within the micro- and nanosockets of the coating surface, whereas positive charges were detected predominantly at the nanorelief peaks. HLPSCs located in the sockets of the CP surface expressed the osteoblastic markers osteocalcin and alkaline phosphatase. The CP multilevel topography induced charge polarity and an EP and overall promoted the osteoblast phenotype of HLPSCs. The negative sign of the EP and its magnitude at the micro- and nanosockets might be sensitive factors that can trigger osteoblastic differentiation and maturation of human stromal cells.

## 1. Introduction

The biological hierarchy from the nanoscale (molecules) to the macrodimensional (organs and organisms) via microsized cells and mesosized tissues clearly indicates that a nano-to-meso-to-macro multilevel approach should be employed for the engineering of biomaterials. Nanoscale engineering is a platform for the “bottom-to-top” approach to the design of biomaterials because organisms communicate with biomaterials at the biomolecule–biomaterial interface.

Modern biomaterial biocompatibility research focuses on stem cell (SC)–biomaterial interactions (that occur at the nano- and microscales) because SCs are the fundamental units that produce/regenerate tissue.

To control SCs, biomaterials mimic the bioimplant surface (BS), with both its morphology and physical/chemical properties being engineered. Only cells adhered to the BS are involved in tissue regeneration when the implant serves as a scaffold.

The influence of surface morphology/roughness on cell attachment has been studied intensively. The significant increase in the number of publications in this area started in 1990, and the number of these publications reached approximately 1100 by April 2017 (SCOPUS, keywords implant-surface-roughness-cell), which is equivalent to 40–55 publications annually.

The physical and chemical properties of a surface are identified by fundamental factors, such as the surface energy (SE), which strongly influences the attachment of cells. The cells adhere to the BS via a specific protein layer that coats the implant shortly after its insertion into a living organism [1]. Fundamentally, attachment of a specific protein molecule obeys adhesion theory [2], which considers dispersion and electrostatic interactions between the particle and the substrate. As the dispersive interaction potential decreases much more quickly with distance *r* (~*r*^−6^) [3] than the electrostatic potential (~*r*^−1^), the the electrostatic potential is expected to have a stronger impact on trapping an electrically charged “tail” of a molecule that travels at some distance from the substrate surface. The electrostatic interaction could be promoted by engineering the electrical charge at the BS. Such an approach is available for dielectric biomaterials, and hydroxyapatite (HAP, a very popular material for implant fabrication) is among them.

The influence of the electrostatic factor on cell attachment has been less explored than the influence of surface morphology/roughness. A significant increase in the number of publications on this topic also started in 1990, and the total number reached approximately 270 by April 2017 (SCOPUS, keywords implant-surface-charge-cell), which is equivalent to 10–13 publications annually.

A few biomaterials are currently in use. However, the present article focuses on the widely exploited biomaterial calcium phosphate (CP). The surface layer electrical charge of CP can be engineered by radiation [4], electrical polarization [5], doping, and reconstruction of the surface atomic/molecular coupling [6]. The surface electrical charge of CP nanoparticles, which are sometimes used to construct an implant, depends on the particle radii [6]. In this connection, the surface roughness, having peaks and valleys that are characterized by the radii of its building blocks, could influence the surface charge.

The surface morphology of CP is typically engineered because there is a set of methods for forming CP coatings, such as sol-gel, plasma spraying, magnetron sputtering, and detonation gas spraying methods. However, these methods have a number of disadvantages. One of the most significant disadvantages is the absence of chemical bonds between the formed coating and the substrate surface. Moreover, the process is long, and the cost of the final product is high.

The abovementioned methods are “direct vision” methods; therefore, they are not used for applying CP coatings to implants. Microarc oxidation (MAO) is more convenient and effective for producing CP coatings on implants with complex shapes and produces good physical and chemical properties [7,8].

The electrolyte (components, pH, and temperature) and the electrophysical parameters of microarc oxidation, such as the anode and anode-cathode modes, voltage, current density, pulse period frequency, time period, and material type (treatment, surface roughness, and electroconductivity) affect the physical, chemical, and biological characteristics of the coating.

A set of key surface physicochemical parameters that affect cells has already been identified, namely, surface mechanical integrity, the rate of scaffold degradation, fluid transport, cell-recognizable surface chemical properties, the ability to induce signal transduction [9], surface free energy, wettability [10], surface electrical charge [11] and chemical composition [12], and surface structure, morphology, and stiffness [13,14].

There is evidence in favor of the influence of HAP surface charge on interactions with biomolecules and cells. The electrical charge deposited on a HAP surface alters its wettability and influences the absorption of proteins [4]. Research reported by [15] demonstrated that a HAP surface electrical potential (EP) shift of 0.5 V influences osteoblastic cell attachment.

In 1964, Curtis and Varde [16] assumed that surface topography has the most important effect on cells. Nevertheless, scientists are only just beginning to understand niche–cell interactions [17]. The achieved results are not unequivocal, and different conditions for fabricating scaffoldings have been recognized as possible factors for the varying outcomes [18]. A series of works [19,20,21,22] focused on determination of the relationship between metallic surface roughness and cell-adhesion parameters. No relation was found between the roughness amplitude, *R_a_,* and cell adhesion. The parameter that better discriminates adhesion is the mean distance between asperities (*S_m_*); however, this correlation is insufficiently strict, and several authors [22,23,24] have developed a new parameter called “adhesion power”.

A wide range of cells (fibroblasts, osteoblasts, osteoclasts, nerve cells, stromal SCs, and embryonic SCs, among others) respond to surface micro- [25,26,27,28] and nanotopography [11,17,29], and the topography is the main influencing factor [12].

However, the connection between the multiple extracellular physicochemical events that control and trigger cells, particularly mesenchymal stem cells (MSCs), is still unclear. The electric field plays a crucial role in the effects of ion channels and cellular ζ potentials [30,31,32,33]. Understanding the relationship between the electric charge of the material surface and protein adsorption is a focus in examining the mechanisms of tissue integration with biomaterials [34]. MSCs and osteoblasts respond to surface charge [34,35,36,37,38] and micro- [39] and nanoreliefs [40,41].

The combination of Ti and CP forms a modern material that is widely used for bioimplant design. The electrical charge of CP surfaces affects their wettability [42], which results in molecular adhesion [43]. The microroughness of CP coatings deposited on Ti using the microarc technique has been shown to control mouse bone tissue remodeling in vivo [44] and osteogenic differentiation of human MSCs in vitro [28].

From the above results, both the morphology and electrical properties of a surface appear to control cells. However, from the electrostatic point of view, the morphology of the surface affects its electrical charge density. Thus, the surface morphology and its electrical charge are possibly interconnected. The nanoscale morphology and particularly the sharpness of the surface affect its electric charge [45], which has its own effect on cells.

Nevertheless, the balance between the physical events that control cell behavior is still unclear. Current studies have not connected surface micro- and nanotopography and the electrical charge as joint factors affecting MSC fate.

This work focuses on the connection between CP surface roughness and the EP at the micro- and nanoscale because they are correlated factors that affect human MSC osteogenic differentiation and maturation in vitro.

## 2. Materials and Methods

### 2.1. Substrate Preparation and CP Deposition

Commercially pure titanium (99.58 Ti, 0.12 O, 0.18 Fe, 0.07 C, 0.04 N, and 0.01 H wt %) plates (10 × 10 × 1 mm^3^) were used as substrates for the deposition of CP coatings. The samples were polished with silicon-carbide paper of 120, 480, 600, and 1200 grit. Then, the samples were cleaned ultrasonically with an Elmasonic S10 device (Elma Schmidbauer GmbH, Siegen, Germany) for 10 min in distilled water immediately before deposition.

Bilateral CP coatings were prepared in the anodal regime as described previously [46] using a Micro-Arc 3.0 apparatus (ISPMS, Tomsk, Russia). The setup consisted of positive and negative pulse power sources, a computer for controlling the deposition process, a galvanic bath with a water cooling system, and electrodes. An aqueous solution prepared from 20 wt % phosphoric acid, 6 wt % dissolved HAP powder, and 9 wt % dissolved calcium carbonate was used to obtain CP. Microarc oxidation was performed in the anodic regime with initial current densities in the range of 0.2–0.25 A/cm^2^. In previous work [47], the optimal microarc oxidation parameters for the deposition of a CP coating on titanium were a pulse frequency of 100 Hz, a pulse duration of 50 μs, and a deposition time of 5–10 min. Samples with different CP coating thickness and CP adhesion strength to the substrate were prepared by varying the electrical voltage in the range of 150–400 V. CP coatings with a thickness of 10–150 μm and a roughness *R_a_* of 1.5–6.5 μm were deposited by varying the electrical voltage and deposition time.

The specimens were dried in a dry-heat manner with a Binder FD53 oven (Binder GmbH, Tuttlingen, Germany) at 453 K for 1 h.

To measure the adhesion strength of the CP coating to the substrate, two cylinders were glued with Loctite Hysol 9514 glue to both sides of the sample with the coating. They were fixed in grips for testing under tension in an Instron 1185 machine (Instron-1185, Buckinghamshire, UK). The adhesion strength was considered to be the maximum stress required to tear the cylinders of the CP coating and was determined by σ = *F*/*S*, where *F* is the breakout force and *S* is the separation area.

### 2.2. Surface Characterization

#### 2.2.1. Surface and Microstructure Observation and Phase Composition

The surface topography and elemental composition of the coating surface and cells were analyzed via scanning electron microscopy (SEM, Philips SEM 515, Amsterdam, The Netherlands) using a microscope equipped with an energy-dispersive X-ray spectroscope (EDAX ECON IV). The surface area was randomly examined at magnifications ranging from 500 to 10,000×. The microstructure of CP coatings was investigated using transmission electron microscopy (TEM, FEI Tecnai 20, Hillsboro, OR, USA). The phase composition was determined by X-ray diffraction (XRD, Bruker D8 Advance, Billerica, MA, USA) in the angular range of 2θ = 5–90° with a scan step size of 0.010° and Cu K*_a_* radiation.

#### 2.2.2. Microroughness Measurements

The substrate surface roughness was assessed with a Talysurf 5–120 profilometer (Taylor Hobson Ltd., Leicester, UK). Measurements of roughness amplitude parameters and the mean value of the profile element width, *S_m_*, were carried out according to the standards ISO 4287-1997 and ISO3274-1996, respectively [48]. Ten randomly selected traces were recorded for each specimen. The average roughness (*R_a_*), peak-to-valley roughness (*R_z_*), and maximum roughness (*R_max_*) were estimated. A strong linear correlation (*r* = 0.95; significance 99%) was identified between *R_a_* and *R_z_* or *R_max_*. Therefore, only R_a_ was used for further roughness characterization.

#### 2.2.3. Optical Microscopy and SEM

An Olympus GX-71 inverted metallographic microscope (Olympus Corporation, Tokyo, Japan) equipped with an Olympus DP 70 digital camera was used to obtain dark field images of the coating relief and to locate alkaline phosphatase (ALP)- or osteocalcin (OCN)-stained cells. In addition, valleys and sockets were analyzed on the CP coatings. Magnifications of 500× and 1000× were applied. Cell morphology and size were demonstrated via SEM (LEO EVO 50, Zeiss, Oberkochen, Germany). Cells were prepared for SEM as described previously [28].

#### 2.2.4. Atomic Force and Kelvin Probe Microscopy

Atomic force microscopy (AFM) was applied in contact mode to characterize the nanoscale roughness of the samples. The average nanoroughness, *R_an_*, was measured. Kelvin probe force measurements were used to identify the surface potential at the micro/nanoscale. A Solver–PRO47 microscope (NT-MDT Co., Zelenograd, Russia) was used for both AFM and Kelvin probe force measurements.

### 2.3. Surface EP

#### 2.3.1. Surface Electric Charge at the Macroscale

The method of lifting the electrode (the Eguchi method) [49] was used to measure the macroscale EP of the surface. The measurements were carried out under ambient conditions. A homemade device was used and is described in detail in [50]. The device measures the electric field potential of weakly charged bodies. The longitudinal resolution of the device was 5 mm, and the measured potentials ranged from tens of millivolts to hundreds of volts. An electrode installed on the surface of the coating was used to measure the charge. The potential induced at the measuring electrode, $V_{in}$, is related to the potential of the surface, $V_{L}$, by the expression:

(1) $$V_{L}=\frac{C_{in}+{С}_{l}}{{С}_{l}}V_{in}$$

where *C_in_* is the input capacitance of the measuring instrument and *C_l_* is the measured capacitance.

#### 2.3.2. Electron Work Function

Because the *V_L_* measurements were performed in air, the voltage could be affected by the environment, and some results appeared dubious. To overcome these doubts, the electron work function (φ) of the specimen surface was estimated based on photoelectron emission detection under high-vacuum conditions.

The value of φ is the minimal energy required for an electron to escape from a solid. The electrical field induced by the surface electrical charge contributes to the value of φ, which increases when the charge is altered in the negative direction. An increment in φ (Δφ) is an index for the surface EP shift.

The specimens were irradiated by soft ultraviolet (UV) light at 3–6 eV (the range of the expected value of φ) to release an electron, and the photoelectron emission current (*I*) was measured. The spectrometer used is described in detail in [51]. The value of φ was identified as the energy of the photons when *I* = 0.

### 2.4. Biological Testing

Before biological testing, the samples were dry-heat sterilized as described above.

#### 2.4.1. Cell Culture and In Vitro Staining

(Human cell isolation and cultivation was permitted by the Local Ethics Committee, Innovation Park, Immanuel Kant Baltic Federal University (permit no. 7 from December 9, 2015).)

Adult adipose-derived MSCs (AMSCs) were isolated from human lipoaspirates of healthy volunteers [52]. The cells were stained using a Phenotyping Kit, human (130-095-198) and Viability Fixable Dyes (Miltenyi Biotec, Bergisch Gladbach, Germany), and the results were analyzed with a MACS Quant flow cytometer (Miltenyi Biotec, Bergisch-Gladbach, Germany) and KALUZA Analysis Software (Beckman Coulter, Brea, CA, USA) according to the manufacturer’s instructions. More than 99% of the viable cells expressed CD73, CD90, or CD105 markers and did not display CD45, CD34, CD20, or CD14 markers (less than 1%).

To confirm the morphofunctional nature of AMSCs, 5 × 10^4^ cells/mL were cultivated in 1.5 mL of medium with reagent from a StemPro^®^ Differentiation Kit (Thermo Fisher Scientific, Waltham, MA, USA) for 21 days, and the medium was exchanged every 3–4 days. After removal of the supernatants, the adherent cells were dried, fixed, and stained with the dyes. According to the recommendations of the International Society for Cellular Therapy (ISCT) and the International Federation for Adipose Therapeutics and Science (IFATS) [52], the multipotent behavior of AMSCs was estimated by cell staining with alcian blue (Sigma-Aldrich, St. Louis, MO, USA), which enabled visualization of proteoglycan synthesis by chondrocytes; alizarin red S (Sigma-Aldrich, St. Louis, MO, USA), which identified mineralization of intercellular substances by osteoblasts; and oil red (Sigma-Aldrich, St. Louis, MO, USA), which detected neutral triglycerides and lipids in adipocytes. All staining was performed as recommended by the manufacturer. The results were assessed with a Zeiss Axio Observer A1 microscope (Carl Zeiss Microscopy, LLC, Thornwood, NY, USA) using ZEN 2012 software (Carl Zeiss Microscopy, LLC).

To determine the effect of CP coatings on AMSC osteogenic differentiation, cells were cultured in an incubator (Sanyo, Japan) at 100% humidity with 5% carbon dioxide at 37 °C for 21 days (the medium was replaced with fresh medium every 3–4 days). The cell suspension was freshly prepared at a concentration of 1.5 × 10^5^ viable cells /mL in 1.5 mL of the following culture medium: 90% Dulbecco’s modified Eagle’s medium (DMEM)/F12 (1:1) (Gibco Life Technologies; Grand Island, NY, USA), 10% fetal bovine serum (Sigma-Aldrich, St. Louis, MO, USA), 50 mg/L gentamicin (Invitrogen, Carlsbad, CA, USA), and sterile L-glutamine solution freshly added to a final concentration of 280 mg/L (Sigma-Aldrich). A titanium substrate with a two-sided microarc CP coating was placed in a plastic well of a 12-well (well area of 1.86 cm^2^) flat-bottom plate (Orange Scientific, Braine-l’Alleud, Belgium).

To establish the self-differentiation potency of cells on a rough CP surface, the culture medium was not saturated by osteogenic supplements. The cells were cultured in DMEM/F12 culture medium either with or without the tested samples (cells were seeded on the samples and around them) and in osteogenic medium from a StemPro^®^ Differentiation Kit (Thermo Fisher Scientific, Waltham, MA, USA). The osteoblasts in cell cultures were stained with alizarin red S as described above. Digital images of AMSCs cultured on CP coatings were obtained via reflected light microscopy on an Olympus GX-71 metallographic device (Olympus Corporation, Tokyo, Japan). ).

Prenatal stromal cells from the human lung (HLPSCs) with a CD34^−^CD44^+^OCN^−^ immunophenotype (Stem Cell Bank Ltd., Tomsk, Russia) were used to study MSC osteogenic differentiation and maturation induced by the nanoscale EP and the roughness of the CP coatings. HLPSCs react to the microroughness of the microarc CP surface according to our previous in vitro investigation [28]. The details are provided in [53]. After the cells were thawed, the viability (92%) of the cells was determined with an ISO 10993-5 test; 0.4% trypan blue was used. One CP-coated specimen was placed in each plastic well of a 24-well flat-bottom plate (Orange Scientific, Braine-l’Alleud, Belgium). The HLPSC suspension was freshly prepared at a concentration of 3 × 10^4^ viable cells /mL in the following culture medium: 80% DMEM/F12 (1:1) (Gibco Life Technologies; Grand Island, NY, USA), 20% fetal bovine serum (Sigma-Aldrich, St. Louis, MO, USA), 50 mg/L gentamicin (Invitrogen, UK), and L-glutamine sterile solution freshly added to a final concentration of 280 mg/L (Sigma-Aldrich). To determine the osteogenic potency of a rough CP surface, the culture medium did not contain osteogenic supplements, such as β-glycerophosphate, dexamethasone, and ascorbic acid. The cell suspension was added to a volume of 1 mL per well. The cells were incubated for 4 days in a humidified atmosphere of 95% air and 5% CO_2_ at 37 °C.

The in vitro viability of adherent cells cultured with the CP coating was estimated with a Countess^TM^ Automated Cell Counter (Invitrogen, Carlsbad, CA, USA) after staining with 0.4% trypan blue (Invitrogen, USA). The percentage of viable and dead (stained) cells was measured after they were harvested with 0.05% trypsin (PanEco, Moscow, Russia) in 0.53 mM EDTA (Sigma-Aldrich, St. Louis, MO, USA) and washed twice with phosphate buffer.

#### 2.4.2. Cytochemical Staining of HLPSCs for ALP via the Diazocoupling Technique

CP-coated titanium specimens with adherent stromal cells were air-dried, fixed for 30 s in formalin vapor, and stained with ALP. Naphthol AS-BI phosphate (C_18_H_15_NO_6_P, molecular weight (m.w.) 452.21) and fast garnet GBC salt (C_14_H_14_N_4_O_4_S, m.w. 334.35) (both from Lachema, Brno Czech Republic) were used. Brown sites indicating enzymatic activity were considered positive cellular ALP-staining [53].

#### 2.4.3. Immunocytochemical Detection of OCN in HLPSCs

Other CP-coated titanium plates were fixed in formalin vapor as described above. Primary antibody (rabbit polyclonal anti-human IgG (1:100), Epitomics Inc., Burlingame, CA, USA) targeting OCN and universal immunoperoxidase anti-rabbit and anti-mouse polymer (Histofine Simple Stain MAX PO MULTY, Nichirei Biosciences Inc., Tokyo, Japan) were used as described previously [52]. The appearance of brown sites in colored cells indicated positive OCN staining.

#### 2.4.4. Computer Morphometry

A morphometry method was used to quantitatively determine cell parameters by measuring their optical features [54]. ImageJ v. 1.43 (http://www.rsb.info.nih.gov/ij) was employed to process digital images of OCN- or ALP-stained cells. Ten randomly selected images were analyzed for each sample. The dimensions of an area of stained cells were calculated (in μm^2^); in addition, the areas (μm^2^) of valleys and sockets that were cell-free or seeded with stained cells were measured on the CP surface.

The area of all valleys in each image was recomputed as a percent of the full image area, and the average fractional area (in %) of valleys (*S_V_*) was calculated and is shown in for each sample with a unique average *R_a_*.

### 2.5. Statistical Analysis

Correlation and regression analyses are provided; the coefficients (*r*) were kept at a significance level higher than 95%.

## 3. Results

### 3.1. AMSC and HLPSC Response to CP Coating

**Figure 1:**
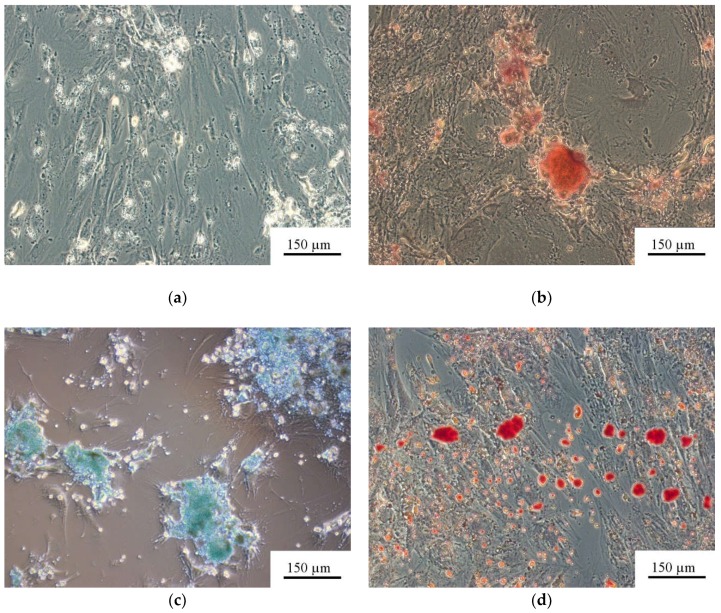
Human adipose-derived mesenchymal stem cells (AMSCs) cultured for 21 days in either (**a**) standard or (**b**–**d**) StemPro^®^ differentiation media. (**a**) Some cells cultivated in the standard medium contained light fatty inclusions (unstained); (**b**) osteogenic medium, alizarin-red-stained area of mineralized intercellular regions; (**c**) chondrogenic medium, alcian-blue-stained glycoproteins; and (**d**) adipogenic medium, oil-red-stained neutral triglycerides and lipids.

Adult (postnatal) fibroblast-like CD73CD90CD105^+^ AMSCs cultivated on plastic wells in a StemPro^®^ Differentiation Kit (Thermo Fisher Scientific, USA) for 21 days were positively stained with alizarin red S (osteoblasts), alcian blue (chondrocytes), and oil red (adipocytes) (Figure 1b–d) and compared with unstained cells in standard medium (Figure 1a). The findings showed that the cells met the morphological criteria of multipotent MSCs (MMSCs) as described in [52].

**Figure 2:**
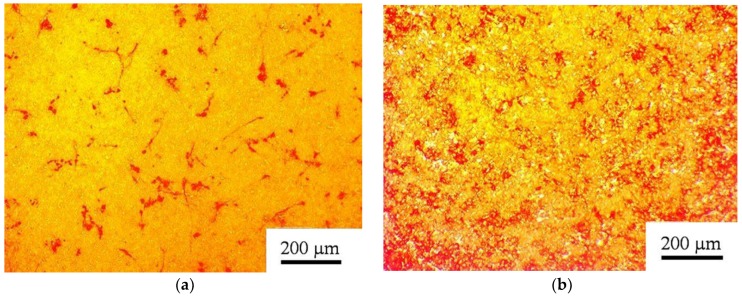
Human AMSCs cultured on a microarc calcium phosphate (CP) coating for 21 days in either (**a**) standard DMEM/F12 or (**b**) osteogenic differentiation media. Alizarin-red-stained areas of mineralized regions of the fibroblast-like cells and their intercellular matrix are shown.

The AMSCs in direct contact with microarc CP coatings in osteogenic supplement-free DMEM/F12 showed in vitro differentiation into osteoblasts (Figure 2a). Thus, a rough CP surface promotes the osteogenic potency of adult AMSCs. The osteogenic medium from the StemPro^®^ Differentiation Kit (Thermo Fisher Scientific, USA) enhanced the osteogenic influence of the CP coating (Figure 2b; red staining). The total area of the alizarin-red-stained cells was 64,500 μm^2^ for AMSCs on the CP surface in standard DMEM/F12 media (Figure 2a) and 249,500 μm^2^ in osteogenic media (Figure 2b).

No cytotoxicity of the CP coating was observed. Initial (before contact with samples) AMSC and HLPSC viabilities (based on the absence of trypan blue staining) were 95% and 92%. Significant differences (*p* <0.05) in the number of viable cells were not observed for the groups studied according to a Mann–Whitney U-test (Table 1).

### 3.2. Coating Phase Composition and Morphology

**Figure 3:**
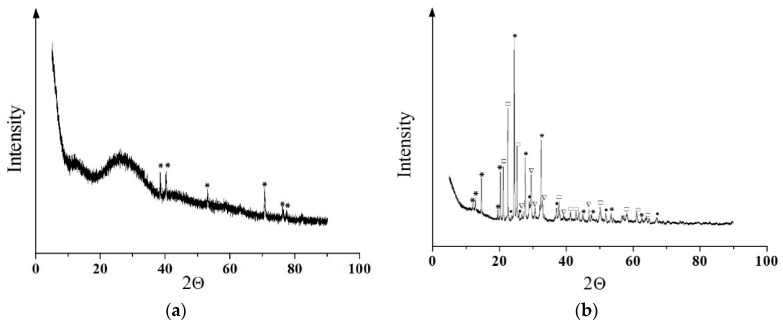
Diffractograms of the CP coating on a titanium surface obtained using the microarc oxidation (MAO) method. (**a**) The amorphous halo from the CP coating and the peaks from the titanium substrate; (**b**) the calcium phosphate coating after annealing at 1073 K. Peaks correspond to the phases: *—CaTi_4_(PO_4_)_6_, □—TiP_2_O_7_, ▽—β-Ca_2_P_2_O_7_, and ●—TiO_2_ (anatase).

**Figure 4:**
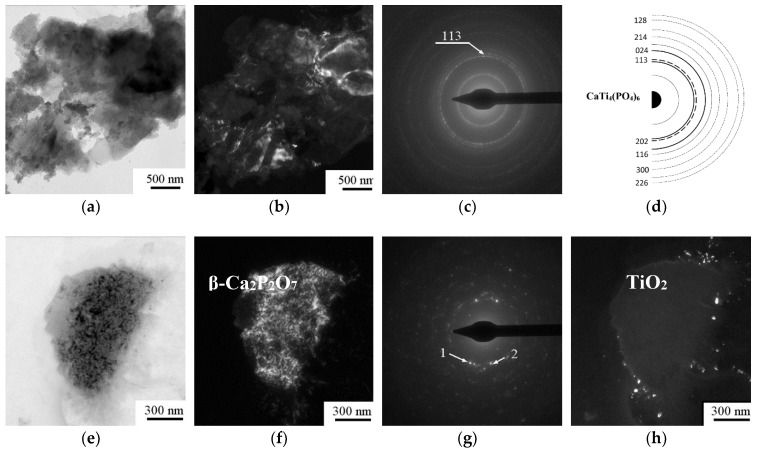
TEM bright field image (**a**,**e**) and corresponding selected area diffraction (**c**,**g**), dark field image (**b**,**f**,**h**), and scheme showing the interpretation (**d**) of the microarc CP coating on titanium after annealing at 1073 K.

XRD (Figure 3) and TEM (Figure 4) data showed that CP coatings deposited on a Ti surface by microarc oxidation are in the X-ray amorphous state, which was confirmed by the presence of a diffuse broad halo. Due to that fact, we were unable to identify the coating phase composition (Figure 3). In addition to the diffuse halo, peaks from the substrate and single CP-associated peaks were identified in the diffractograms. The crystallite size observed in the dark field images was 10–20 nm.

Samples were annealed in air at 1073 K for 1 h to fully identify the phase composition. The main phase of the coating was CaTi_4_(PO_4_)_6_ with a fraction of β-Ca_2_P_2_O_7,_ TiP_2_O_7_ and TiO_2_ (anatase) (Figure 3b and Figure 4). Mean crystallite sizes were estimated by darkfield analysis: the mean sizes of CP phases reached 60–80 nm and those of oxides were not greater than 30–40 nm.

**Figure 5:**
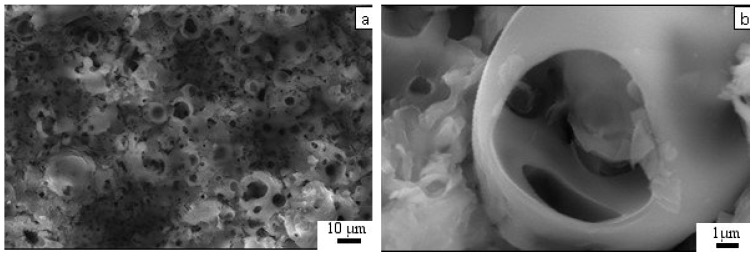
SEM images of the CP coating.

The microreliefs of the CP surface exhibited irregularities. The peaks of the CP microreliefs consisted of spherulites of up to 10–30 µm in diameter (Figure 5). Interconnected valleys are shown in Figure 5a as dark vast fields between ranges of bright spherulites. Single or open interconnected pores (1–10 μm in diameter) were revealed in both spherulites (Figure 5b) and valleys.

**Figure 6:**
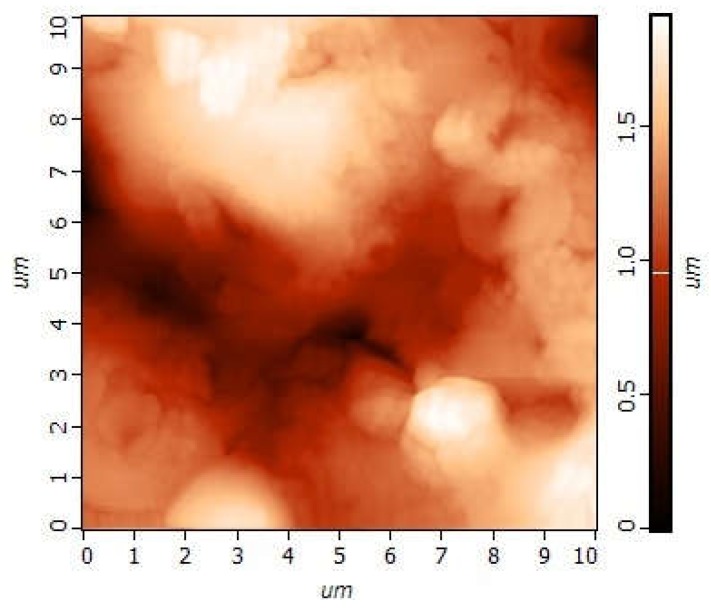
AFM image of the CP surface.

AFM measurements of the CP coating showed that its relief was embedded with 500–1000 nm submicron particles (Figure 6). The particles assembled in globules that were 1–2 μm in diameter and 30 nm in height. The positioning of the globules provided porous (pores of 1–2 μm diameter) microspherulites (3–5 μm diameter, 300 nm in height).

### 3.3. Relationship between Electrostatic, Geometrical, and Cytological Properties of the CP Coating

#### 3.3.1. Microscale

**Figure 7:**
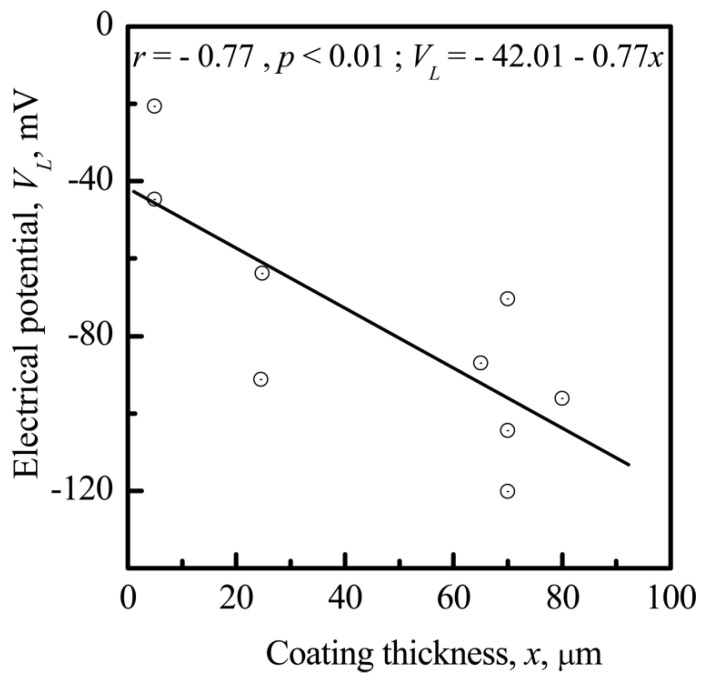
Correlation between the electrical potential (EP), *V_L_*, and the CP coating thickness, *x.*

**Figure 8:**
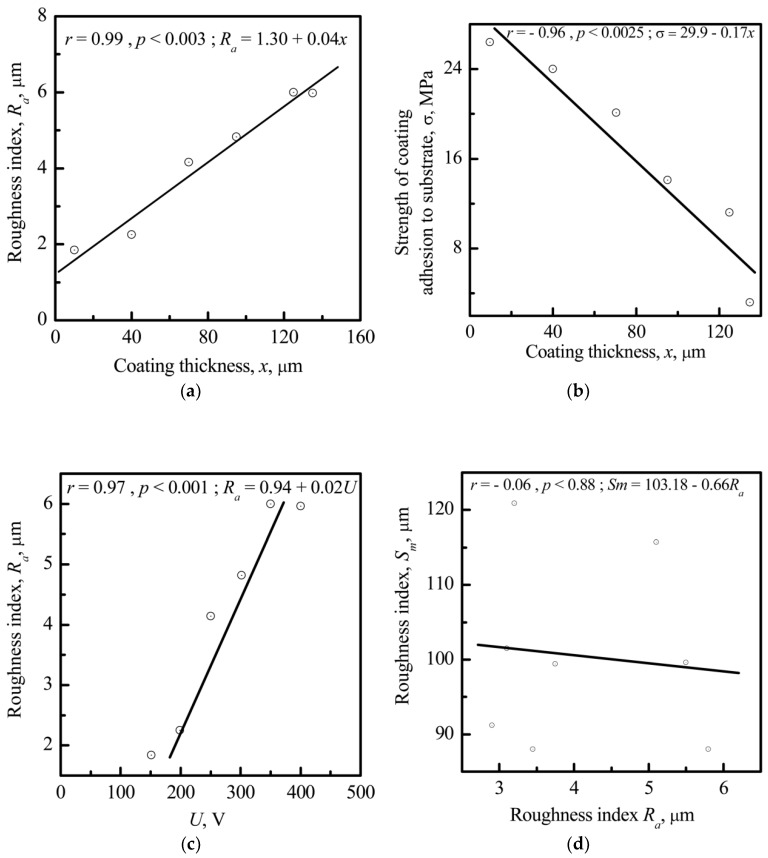
Correlations between (**a**) the CP coating thickness (*x*) and the roughness index *R_a_*; (**b**) the CP coating thickness (*x*) and the adhesion strength of the CP coating to the metal substrate; (**c**) the amplitude of the electrode voltage (*U*) of the microarc device and the roughness index *R_a_*; and (**d**) the CP roughness indices *R_a_* and *S_m_*_._

*V_L_* is correlated with the thickness (*x*) of the CP coatings (*r* = −0.77; significance 99%) (Figure 7). The negative value of *V_L_* increases with increasing *x*. On the other hand, *x* is also correlated with the surface roughness index *R_a_* (*r* = 0.99; significance >99%) (Figure 8a).

At the same time, the adhesion of the microarc-fabricated rough CP coating to the metal substrate decreased directly with increased coating thickness (Figure 8b). Thus, implants with a thick CP coating (thickness ≥40–60 μm, see Figure 8b) were biomechanically unsuccessful. Further, Figure 8c demonstrates a strong direct correlation (*r* = 0.97; significance >99%) between the amplitude of the electrode voltage (*U*) of the microarc device and the roughness index *R_a_*.

No correlations were observed between *S_m_* and *R_a_* (Figure 8d; *r* = −0.06; significance >88%) or other CP coating parameters (thickness, *V_L_, R_z_*, and *R_max_*). Therefore, only the roughness index *R_a_* (and not the index *S_m_*) may be controlled technologically during CP coating via microarc deposition.

Surface microtopography is biologically necessary for osteoblasts [20] to stimulate bone tissue regeneration. Previously, a microarc-fabricated rough CP coating has exhibited osteogenic activity in vivo [11,44] and in vitro [28]. Therefore, a good connection between the CP surface roughness index *R_a_* and *V_L_* is likely important for designing thin coatings with optimal osteogenic and biomechanical cues.

Based on the results described above, a strong correlation between *V_L_* and *R_a_* was sought. However, such a correlation was not found (*r* = −0.33; significance >99%), favoring the conclusion that electrical charge does not depend closely on *R_a_*; however, a surface charge could be induced by *R_an_*, i.e., at the nanoscale [45,55].

**Figure 9:**
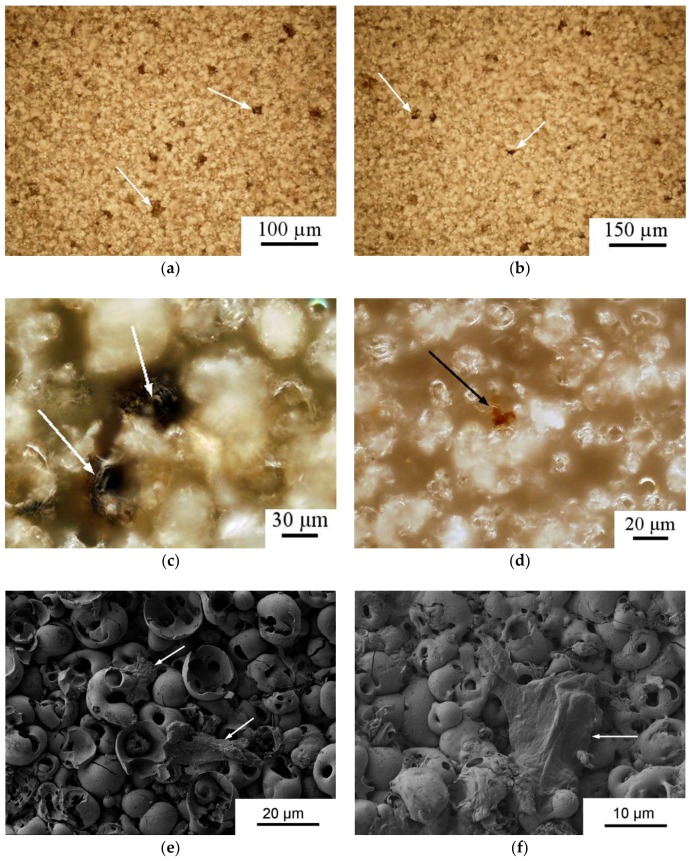
Surface distribution (**a**,**b**) and typical locations of alkaline phosphatase (ALP)- (**a**,**c**) or osteocalcin (OCN)-stained (**b**,**d**) HLPSCs (brown sites) in the sockets of the microarc-fabricated rough CP coating (reflecting optical microscopy) and SEM images of the HLPSCs (**e**,**f**). Cells are indicated with white and black arrows.

The stromal cell–surface interaction plays a dominant role in bone tissue growth. Cells can migrate into pores if the pores are more than 10 μm in diameter. However, the pores of microarc CP coatings are smaller (~1–10 μm), and MSCs likely do not seed in them. As a result, they preferentially adhere to the spherulites and valleys in the CP coating [28]. The valleys consist of single sockets (Figure 5a and Figure 9). HLPSCs located in the sockets of the CP surface expressed the osteoblast markers ALP and OCN (Figure 9a–d). Approximately 84% of the ALP- or OCN-positive cells were found in the CP surface sockets, and only 16% were found on the spherulites.

According to SEM, HLPSCSs are approximately 20–25 µm. EDX measurement of cell-like sites on the CP surface shows the elemental composition (78.63–79.16 C, 16.03–16.45 O, 2.84–2.94 P, 0.70–0.71 Ca, 1.26–1.28 Ti atomic %), which is unlike that of the CP coating itself (55.84 O, 25.22 P, 5.34 Ca, 13.60 Ti atomic %). Therefore, Figure 9e,f shows cells on the relief of the CP surface.

**Figure 10:**
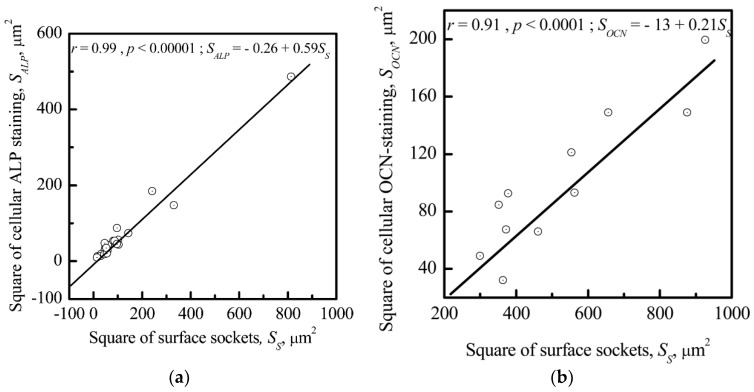
Correlations between the areas containing ALP-stained (**a**) *S_ALP_* or OCN-stained (**b**) *S_OCN_*. HLPSCs and the surrounding sockets (*S_S_*) of the CP surface.

Figure 10 shows a strong correlation between the areas of ALP- (*r* = 0.99; significance >99%) or OCN-stained cells (*r* = 0.91; significance >99%) and the surrounding socket areas seeded with HLPSCs. Thus, the CP microrelief promotes an in vitro cell osteoblastic phenotype without osteogenic stimulators (β-glycerophosphate, dexamethasone, and ascorbic acid) in the culture medium.

**Figure 11:**
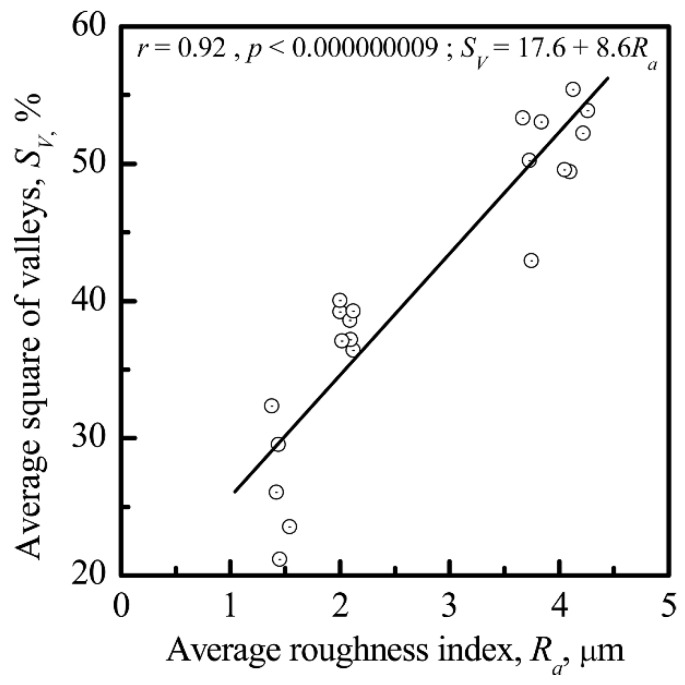
Correlation between the average area of valleys (*S_V_*) and the average roughness index (*R_a_)* of the CP surface.

**Figure 12:**
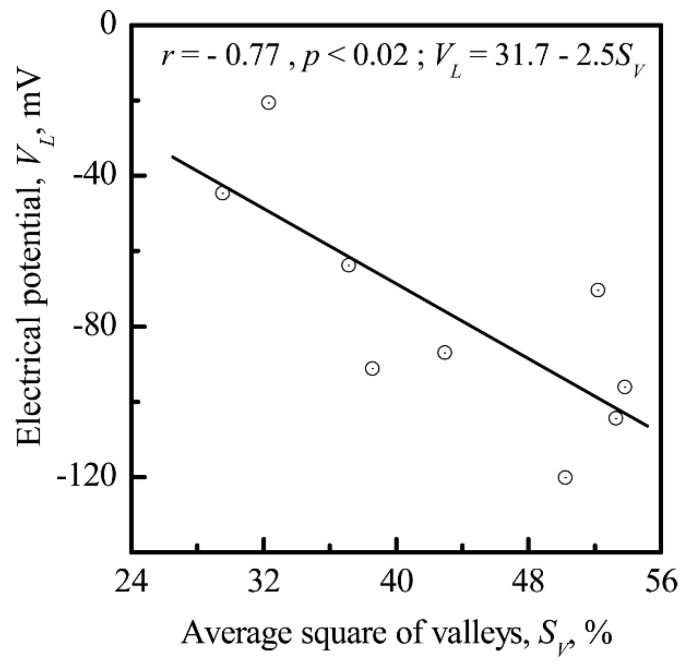
Correlation between the EP (*V_L_*) and the average area of valleys (*S_V_)* on the CP surface.

HLPSCs were attracted to the valleys and sockets of the microarc CP coating. The *R_a_* of the CP surface had an effect on the *S_V_* of the coating (*r* = 0.92; significance >99%) (Figure 11). *V_L_* was also correlated with *S_V_* (*r* = −0.77; significance 98%) (Figure 12).

The surface valleys were formed by microsockets (Figure 5 and Figure 9), and their areas (*S_V_* and *S_S_*) were correlated (*r* = 0.92; significance >99%). In addition, the correlation between *V_L_* and *S_S_* for rough CP surfaces was rather high (*r* = –0.79; significance >99%) (not shown).

#### 3.3.2. The Nanoscale

**Figure 13:**
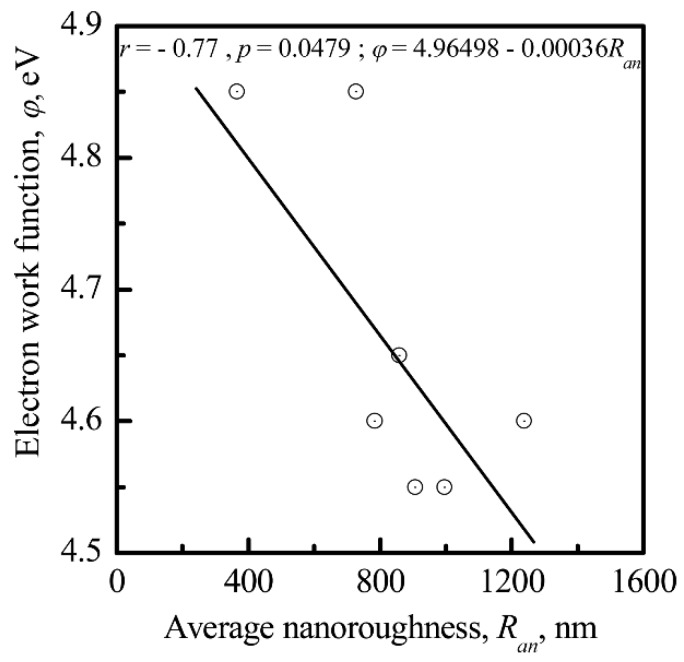
The dependence of the electron work function (*φ*) on the average nanoroughness (*R_an_*) of the CP coating.

**Figure 14:**
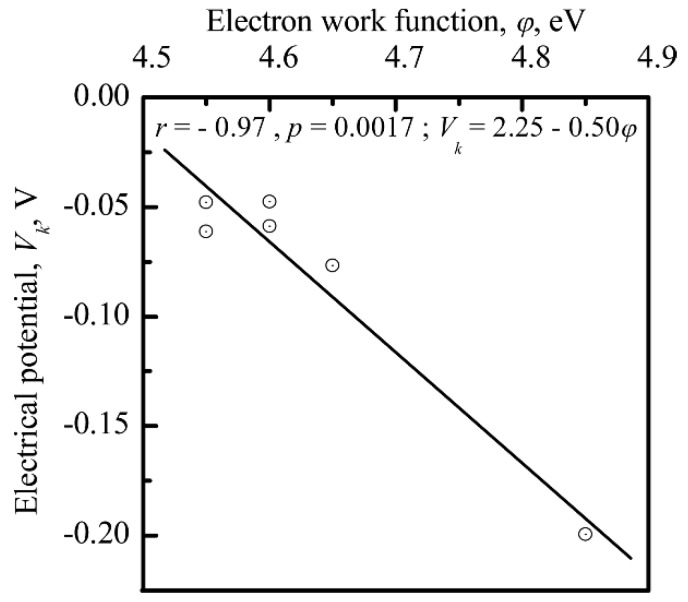
Relationship between the EP (*V_k_*) and the electron work function (*φ*) of the CP surface at the nanoscale level.

The value of φ was correlated with *R_an_* (*r* = −0.77; significance 95%; Figure 13), and the nanoscale potential (*V_k_*) was correlated with *φ* (*r* = −0.97; significance 95%; Figure 14) for the CP surface, supporting the finding that the electrical charge density was controlled by the surface relief at the nanoscale. The sharpness of the surface topography (peaks and sockets) might be the factor that affected the electrical charge density of the CP coating.

A strong correlation between the surface electrical charge density and *R_an_* (Figure 13 and Figure 14) could explain the impact that both the electrical charge and the surface morphology of the microarc CP coating had on cell osteogenic activity.

**Figure 15:**
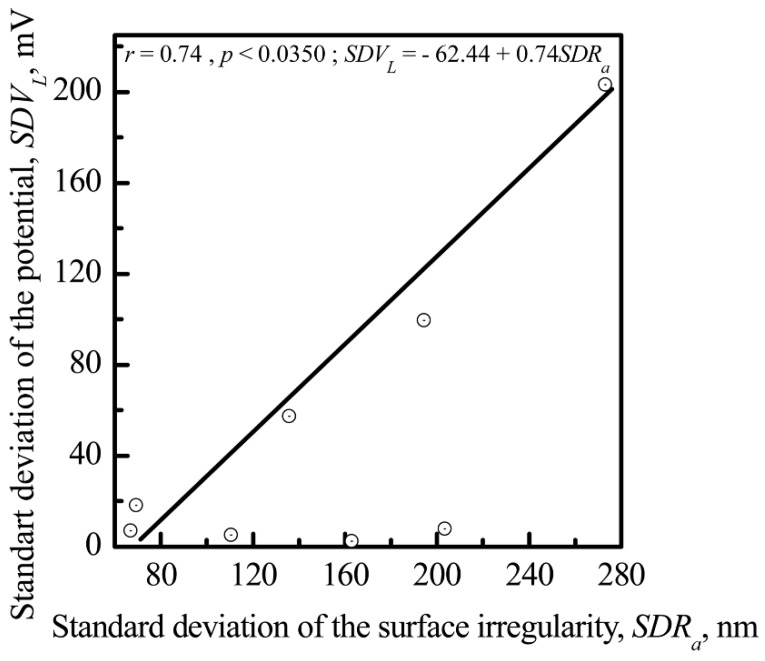
Correlation and interconnection of the standard deviations of surface potential (*SDV_L_*) and surface irregularity (*SDR_a_*) for the CP surface.

The above microscopy results (Figure 6) demonstrate that the surface nanotopography was constructed from nanosized and submicron particles. The average size of the particles was similar for all specimens. Therefore, the deviation in CP coating irregularities was estimated as the standard deviation of *R_a_* (*SDR_a_*). Following the above results, a correlation of *SDR_a_* with the standard deviation of the surface EP (*SDV_L_*) was obtained (Figure 15). The results demonstrated a strong dependence of *V_L_* standard deviation on the *R_a_* standard deviation for microarc CP coating.

**Figure 16:**
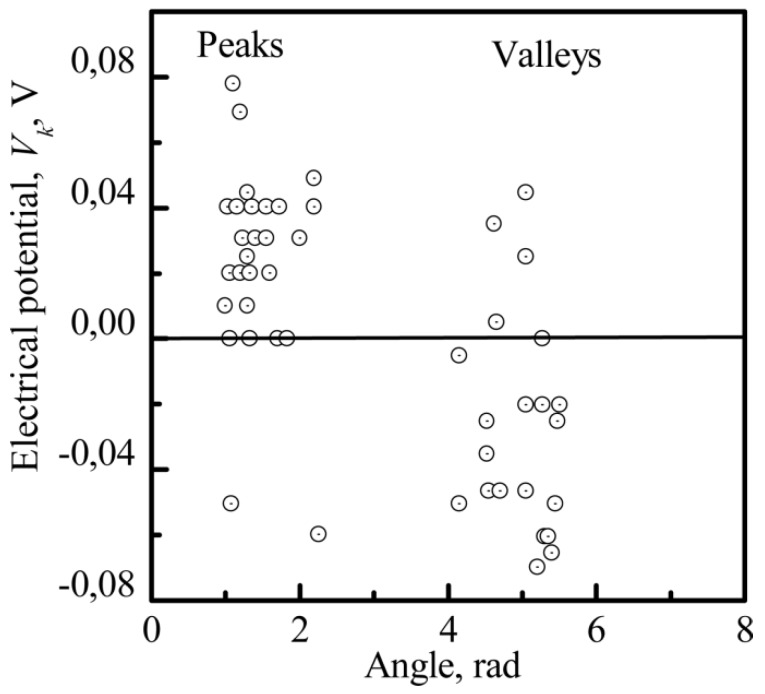
The distribution of the EP of the CP surface (*V_k_*) versus the vertexes and interior angles of the CP nanorelief.

The sharpness of a peak can be characterized by its apex angle. The sharpness value gives an indication of the electrical field delivered by the surface electrical charges (according to general electrostatics, a higher sharpness value indicates a stronger electrical field). Figure 16 demonstrates the distribution of *V_k_* measured by the Kelvin probe method. *V_k_* is more positive at the peaks of the CP nanorelief than at the valleys.

## 4. Discussion

Cells are immobilized in vivo within tissue and bound on a diverse array of scaffolds that are considered the extracellular matrix (ECM) of the native microenvironment [17]. The specific microenvironment where SCs exist is called the SC niche (SCN). Knowledge of the SCN and ECM control of cell fate provides tools for stimulating SC differentiation into desired cell types [29]. Biomimetic ex vivo modeling of SCNs by means of artificial materials has been attempted, and the cell behavior in such foreign ECMs has been studied [56,57,58]. Bone is well-known to be a native substrate for marrow MSCs, and the surface topography of the mineralized bone surface essentially affects cell fate [56]. MSCs serve as the determining component for controlling haematopoietic SCs [59].

Currently, CP surfaces have been used to imitate the mineralized bone matrix and are the most advanced ECM model. Earlier experiments have demonstrated that the microroughness of a microarc CP coating has a significant effect on the osteogenic potency of mouse bone marrow MSCs in vivo [44] and human HLPSCs in vitro [28].

MSCs are characterized as follows [52,60]: They (1) exhibit 90% viability; (2) express the surface markers CD73, CD90, and CD105 and do not express the hematopoietic markers CD45, CD34, CD20, and CD14; and (3) attach to plastic and differentiate into osteogenic, chondrogenic, and adipogenic lineages.

The in vitro culture in this work showed that adult (postnatal) fibroblast-like AMSCs positive for CD73, CD90, and CD105 met the morphological criteria for multipotent MSCs (Figure 1). In turn, a rough CP surface promoted the osteogenic potency of adult tissue-specific AMSCs (Figure 2). As a result, the occurrence of so-called “osteoblastic niches” may occur in MMSC culture [59] on CP coatings. However, AMSCs are very large cells with a size of approximately 200 µm, and their large size complicates estimation of fibroblast-like cell location and the staining area on CP relief using SEM and reflected light microscopy. Thus, HLPSCs, which are 20–25 µm, are useful for studying cell surface distribution.

Moreover, HLPSCs use in-vitro-supplied information about the early stages of bone tissue regeneration because of the ability of embryonic SCs (ESCs) to self-organize the SCN for their own development [61]. On the other hand, microenvironmental factors and, particularly, physicochemical factors regulate the self-maintenance/differentiation of ESCs in the same way as they do for tissue-specific SCs [56]. Therefore, similar effects of the nanoscale EP and the roughness of the CP coatings on SCs were studied using HLPSCs. As discussed in previous studies [22,23,24], an original statistical approach was developed for characterizing the relationship between surface roughness and cell adhesion. For two materials, 316L stainless steel and TiAl6V4 titanium alloy, a relationship between *R_a_* and cell adhesion has not been found. Cell adhesion was more related to the roughness organization of substrates than to *R_a_*. A more convenient parameter is the mean value of the profile element width, *S_m_*. However, this correlation was insufficient, and for a more accurate characterization of cell-adhesion and roughness parameters, the new adhesion parameter “adhesion power” has been suggested. All these parameters are related in metallic materials. The situation is different for insulating materials.

The relationship between cell adhesion and roughness for CP coatings is likely more complicated because of the influences of not only surface chemistry and topography roughness organization but also of surface electrical charge, which is related to the dielectric properties of a CP coating. The size of the niche (microterritories for SCs) and its composition regulate the balance between differentiation-inducing and differentiation-inhibiting factors for both ESCs and adult SCs [61]. A quantitative assessment of the effect of CP microroughness on HLPSC differentiation into osteoblasts has led us to speculate that separate osteogenic microterritories for MSCs must exist in the ECM and may be reconstructed artificially [28]. The CP microrelief and electrical voltage have been proposed as key factors in the function of osteogenic niches [62].

However, the significance and interconnection of the CP surface properties (roughness, native charge sign, and EP amplitude) at the micro/nanoscale for human MSC osteogenic differentiation and maturation in vitro are not known.

Microscopic studies at the micro/nanorelief level have been performed on rough CP surfaces (*R_a_* = 1.0–4.5 μm) prepared via the microarc coating technique. According to Figure 5 and Figure 6, the microarc technology produces a three-dimensional (3D) scaffold with a bone-like thick CP coating and a multilevel structure consisting of spherulites, valleys, sockets, and pores. Thus, microarc-based simulation of the architecture of porous bone could be directed toward in vivo remodeling of the bone/bone marrow system [44,63].

Our data correspond to work on another type of CP surface [18,63,64,65,66,67,68]. CP ceramic roughness has specific osteoinductive properties [69].

Knowledge of the structure of osteogenic biomaterials that are identified by morphology and pore structure is necessary for MSC and osteoblast development and bone formation and clearly should be complemented with an understanding of the physicochemical characteristics that are extremely important to biomaterial design. There have been attempts to consider the effect of physicochemical and structural peculiarities on cellular and molecular reactions; however, the general mechanisms are not completely understood [70].

The linear relationship between the microscale surface electrical potential (*V_L_*), thickness (*x*), and roughness (*R_a_*) of the CP coating is shown in Figure 7 and Figure 8.

The roughness spacing parameter *S_m_* characterizes cell adhesion to the raised surface of metallic materials. In our case, no correlations were observed between the roughness index *S_m_* and *R_a_* (Figure 8d) or other parameters (thickness, *V_L_, R_z_*, and *R_max_*) for the microarc CP coating. The roughness index *R_a_*, unlike the *S_m_* index, may be technically controlled during CP coating microarc deposition because of its close connection with the *U* of the microarc device (Figure 8c). Therefore, S_m_ could be used as a parameter related to cell adhesion but only for the particular case of metallic implants.

The connection of *V_L_* and *R_a_* to *x* provides a technological tool to engineer both the roughness and surface EP by controlling the CP coating thickness. This tool could be used to control cell sedimentation, migration, and adhesion to the CP surface. *V_L_* is the factor that affects the distribution of a suspension of cells before their direct contact with a CP surface as identified by the *V_L_* measurement obtained using the electrode lifting approach and revealed by the electrical field approximately 500 μm above the surface [50].

**Figure 17:**
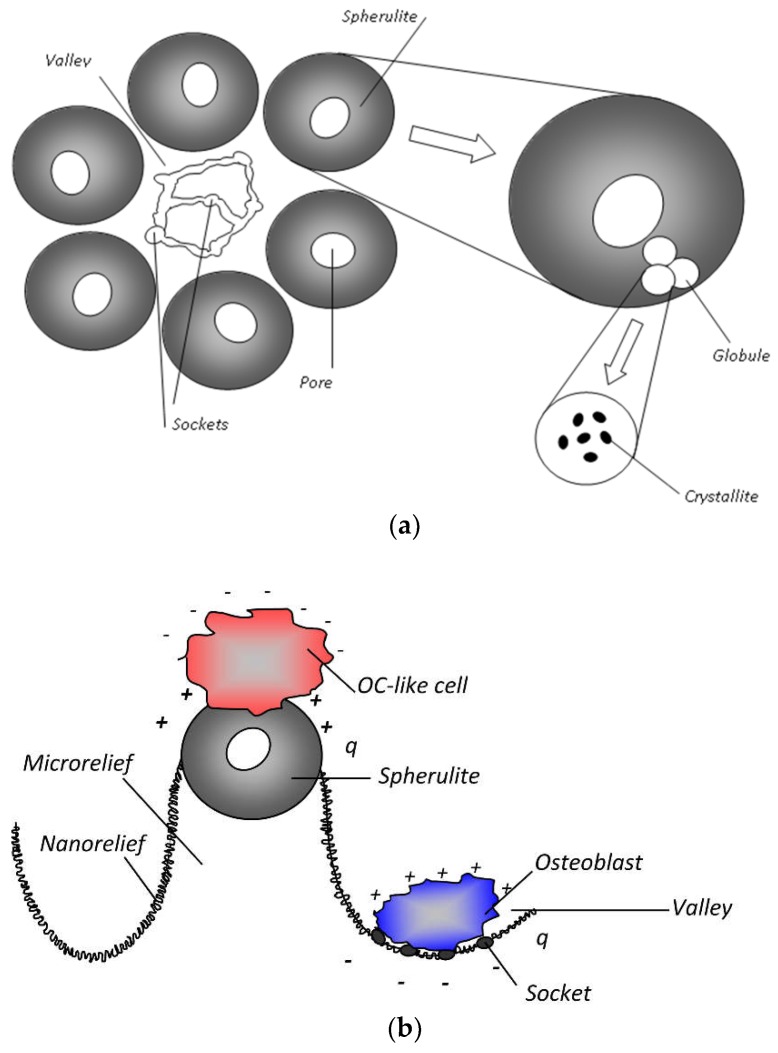
Schematic image of structural elements of the MAO CP coating (**a**) and nonuniform differentiation of cells in HLPSC culture (**b**). OC, osteoclast.

This process must affect the 3D features of sedimentation, distribution, and seeding and the morphofunctional state of human MSCs and HLPSCs, in particular. Different stromal cells present opposite ζ potentials, which are revealed in an external electric field. For instance, the fibroblast surface is typically negatively charged [36], while osteoblasts are characterized with a positive ζ potential [71]. Therefore, fibroblast-like cells stained with acid phosphatase and ALP-stained osteoblast-like cells could be localized at the spherulites and sockets (Figure 17), respectively, as established previously in [28].

The range in which cells are “sensitive” to the size of surface structures is usually very small and corresponds to the nano- and microscale. Detailed knowledge of their effect on cellular behavior has not been attained. A positive surface charge stimulates differentiation of osteoclast-like cells [72]. A direct electrostatic interaction between a cell and the substrate surface is considered to be a predictor of cell adhesion for implants [73]. The morphology of microarc CP coatings and its close correlation with *V_L_* (Figure 7 and Figure 12) could have an effect on HLPSCs, which impacts cellular colonization based on the formation of a cell–surface interface [25]. Osteoblasts respond to substrate microarchitecture [74]. Cells adhere to and proliferate on smooth surfaces (plastics, glass, and titanium) but have relatively low differentiation indices. Their proliferation decreases but differentiation increases when they are cultivated on microrough surfaces with an *R_a_* index of 4–7 μm. Costa et al. [39] recently demonstrated the capability of biomimetic HAP coating topography to influence the attachment and differentiation of osteoblasts and the resorptive activity of osteoclasts. Osteoblast attachment and differentiation were stronger on more complex, microrough HAP surfaces (*R_a_* ~2 μm) than on smoother topographies (*R_a_* ~1 μm). In contrast, osteoclast activity was greater on smooth surfaces than on microrough surfaces. Thus, there are essential differences between two-dimensional (2D) and 3D cell cultures in addition to differences in cell behavior in vivo [75].

Microarc CP rough surfaces are capable of supporting an HLPSC 3D culture in the presence of osteogenic supplements (β-glycerophosphate, dexamethasone, and ascorbic acid) [28,62].

We previously named the sockets on the CP surface populated by ALP-stained (osteoblast marker) stromal cells artificial osteogenic “niches” [62]. Our culture strategy was used for the current experiments; however, osteogenic supplements were absent from the culture medium. Nevertheless, the rough CP coating alone caused HLPSC adhesion in the surface sockets and ALP and OCN expression (Figure 9). The direct dependence of ALP-staining intensity in HLPSCs on the areas of the surrounding sockets was shown (Figure 10). Thus, distinct deep microterritories of CP topography mainly promote the osteoblastic phenotype of HLPSCs.

Surface morphology determines SC attachment, spreading, proliferation, and differentiation [17,29]. However, the underlying mechanisms that trigger SC differentiation are not entirely clear [29]. AFM with a Kelvin probe was used to study the micro- and nanoscale features of the microarc CP coating.

Interconnections between the electrostatic and morphological indices of the microarc CP coating at the micro- (Figure 11 and Figure 12) and nanoscale were discovered (Figure 13, Figure 14 and Figure 15). The nanorelief of the microarc CP coating can define the sign and amplitude of the EP at the micro/nanoscale. Positive charges were located at the peaks of the relief, and negative polarity was observed in the valleys and sockets (Figure 16). The valleys were assembled by the microsockets (Figure 5 and Figure 9), which led to a close correlation of the microscale *V_L_* value with *S_V_* of the surface valleys (Figure 12) and irregularities in the roughness (Figure 11 and Figure 15). This correlation must affect the 3D features of human HLPSC differentiation and maturation at the micro/nanoscale.

Thus, the results revealed a nonuniform distribution of the CP topography (Figure 17a) and the charge and electric voltage values at the micro/nanoscale. We believe that the CP relief affects the surface charge sign and the magnitude of the electric voltage at the CP nano- and microsockets, and all are integrated together as joint physical factors that trigger MSC osteoblastic differentiation and maturation on the microarc CP coating (Figure 17b). The microarc coatings are X-ray amorphous and contain many primitive CP compounds (CaTi_4_(PO_4_)_6_, β-Ca_2_P_2_O_7_, TiP_2_O_7_, TiO_2_ (anatase)) (Figure 3 and Figure 4). One could assume that the polarity of the CP nano/microrelief is linked to the nonuniform CP phase distributions at the structural elements (spherulites and sockets) on the surface.

The electric fields play a crucial role in the fate of SCs because of their effects on cell transmembrane properties (via ion channels) and ζ potentials [30,33]. Nevertheless, the inter- and intracellular interconnections controlling MSC differentiation into osteoblasts are still not clearly understood.

Recent studies have demonstrated that multiple ion channels are heterogeneously present in the membranes of different SCs, including MSCs [33]. Therefore, hyperpolarization plays an important role in differentiation and maturation of both excitable and nonexcitable cell types. Hyperpolarization of the negative membrane potential promotes osteogenic (ALP gene expression and intracellular calcium level) differentiation of human MSCs, unlike when this membrane is depolarized [76,77]. Scaffold topography alters the intracellular calcium dynamics [78] and the state of chloride channels in cultured cells [79].

Therefore, the micro/nanoscale electric charge distribution on the microarc CP surface might affect the endogenous transmembrane potential of human MSCs through voltage-dependent ion channels and protein polarization, enabling control of cellular osteogenic differentiation and maturation. The presented topographical and electrical interconnections of the CP scaffold features are fascinating and contribute to a fundamental understanding of cell fate control in addition to being a potential tool for bone tissue engineering.

## 5. Conclusions

Direct interaction of cells with biomaterial surfaces is the key to surface biocompatibility [25]. Certain parameters correlate cell adhesion [80] and proliferation with the roughness of the substrate surface [10]. The present study revealed close correlations between the CP microscale surface roughness and the surface native charge, polarity, and electric potential magnitude. The negative charge and electric voltage of microarc CP coatings increase with surface thickness. Because of this phenomenon, the electric field should control the sedimentation and distribution of suspended cells before their direct contact with the CP surface.

The coating demonstrated an interconnection between CP nanoroughness and EP. The nanorelief of the microarc CP coatings defined an irregular pattern in the sign and magnitude of the EP. The nonuniform morphology of the microarc coatings led to an accumulation of negative charges within the sockets of the CP coating; however, the peaks were characterized by a positive charge. Unequal voltage distribution at the nanoscale must selectively affect cell attachment and spreading to the CP surface.

It seems that the negative charge and the magnitude of the surface EP at the nano- and microsockets (“artificial niches” [28]) are joint factors affecting the physical mechanisms that trigger MSC osteoblastic differentiation and maturation on a microarc CP coating.

The present work developed an approach involving functionalization of a substrate surface while considering both the micro/nanoscale relief (“niche-relief” concept) and EP (“niche-voltage” concept) [62] for prospective multilevel bone tissue engineering.

## Figures and Tables

**Table 1:** Percentage of viable AMSCs or prenatal stromal cells from the human lung (HLPSCs) after cultivation with a CP-coated surface for 21 or 4 days, Me (Q_1_–Q_3_).

Group Studied (*n* = 5)	AMSC Viability, %	HLPSC Viability, %
Control cell culture	88 (88–94)	91 (88–92)
Cell culture in contact with the CP-coated sample	87 (81–90)	90 (88–91)

Note: Here, *n* = the number of observations (samples) in each group.
